# Avoiding Premature Diagnostic Closure: Lessons from Two Children with Neurotransmitter Disorders Associated with Dual Pathology

**DOI:** 10.1002/mdc3.14164

**Published:** 2024-07-31

**Authors:** Ainara Salazar‐Villacorta, Robert Spaull, Samyami Chowdhury, Bina Mukhtyar, Manali Chitre, Ruth Armstrong, Mario Sa, Saleel Chandratre, Usha Kini, Ravi Chinthapalli, Kshitij Mankad, Sniya Sudhakar, Simon Pope, Simon Heales, Manju A. Kurian

**Affiliations:** ^1^ Neurogenetics Department UCL Queen Square Institute of Neurology London UK; ^2^ Developmental Neurosciences Zayed Centre for Research into Rare Disease in Children, UCL Great Ormond Street Institute of Child Health London UK; ^3^ Department of Neurology Great Ormond Street Hospital London UK; ^4^ Division of Neurology The Hospital for Sick Children Toronto Ontario Canada; ^5^ Department of Paediatrics Norfolk and Norwich University Hospital Norwich UK; ^6^ Department of Paediatric Neurology Cambridge University Hospitals NHS Foundation Trust Cambridge UK; ^7^ Department of Clinical Genetics Cambridge University Hospitals NHS Foundation Trust Cambridge UK; ^8^ Department of Paediatric Neurology Oxford University Hospitals NHS Foundation Trust Oxford UK; ^9^ Oxford Centre for Genomic Medicine Oxford University Hospitals NHS Foundation Trust Oxford UK; ^10^ Department of Paediatrics The Great Western Hospital Wiltshire UK; ^11^ Department of Radiology Great Ormond Street Hospital London UK; ^12^ Neurometabolic Unit The National Hospital for Neurology and Neurosurgery London UK

**Keywords:** neurotransmitter disorders, movement disorder, red flag, global developmental delay, dual pathology, blended phenotype

Inherited disorders of neurotransmitter metabolism comprise an expanding heterogeneous spectrum of diseases associated with complex movement disorders, autonomic features, and global developmental delay (GDD).^1^, ^2^ Monoamine neurotransmitter disorders (NTD) are due to primary or secondary defects in the biosynthesis, degradation or transport of dopamine, norepinephrine, epinephrine, and serotonin. Here, we describe two patients where the diagnosis of a treatable NTD was confounded by the presence of dual pathology.

## Case Report

**Patient 1**, an adopted 9‐year‐old girl, was born at full‐term by normal delivery, following normal pregnancy. She presented at 2 years with GDD. A maternally inherited microdeletion 14q22.2‐q22.3 (1.8 Mb, GRCh37 (54674679‐56476574) × 1) including the *GCH1* gene was identified via chromosomal microarray; similar deletions had been linked to complex developmental syndromes.^3^, ^4^ The mother had learning difficulties and attended a special school, but was not reported to have motor symptoms. Given the uncertainty surrounding the contribution of *GCH1* to the phenotype, close pediatric monitoring was advised, but the patient was lost to follow‐up following adoption.

She re‐presented at 8 years, with progressive gross motor difficulties, a high stepping gait, and bilateral foot inversion that worsened during the day. She had additional cognitive and fine motor difficulties and had developed progressive scoliosis. Facial dysmorphia (hypertelorism, downward slanting palpebral fissures), hypotonia, joint hypermobility and action dystonia of the upper and lower limbs were present (Video 1, part 1). Magnetic resonance imaging (MRI) showed a focal cortical migration anomaly (Fig. S1) and cerebrospinal fluid (CSF) neurotransmitters showed an abnormal pterin profile consistent with *GCH1* deficiency (Table 1). Sinemet (levodopa [l‐dopa]/carbidopa) was started with significant improvement of motor symptoms. At 9 years old, there was marked amelioration of gait (Video 1, part 2). She also reported increased exercise tolerance and better energy levels.

**Video 1:** Clinical features of patient 1 before and after treatment with levodopa

**TABLE 1:** CSF neurotransmitter values in the reported patients with age‐related normal reference ranges

	Patient 1	Patient 2	Age‐related reference ranges
HVA	187 nmol/L	159 nmol/L^a^	71–565 nmol/L
5‐HIAA	110 nmol/L	166 nmol/L^a^	58–220 nmol/L
HVA:5HIAA ratio	1.70	1.00	1.0–3.7
5‐MTHF	**61** nmol/L	**39** nmol/L^b^	72–172 nmol/L
BH2	1.0 nmol/L	**57.2** nmol/L	0.4–13.9 nmol/L
BH4	**<4** nmol/L	28 nmol/L	9–39 nmol/L
Total neopterin	**5** nmol/L	30 nmol/L	7–65 nmol/L
Pyridoxal phosphate	**44** nmol/L	26 nmol/L	10–37 nmol/L

*Note*: At the time of the analysis, Patient 1 was 8 years old, and treatment had not yet been started, Patient 2, on the other hand, was 9 years old and treatment was already initiated. **Abnormal values highlighted in bold**. Patient 1 pterin profile is typical of *GCH1*‐deficiency. HVA and 5‐HIAA are often, but not always, as in our patient, below the lower limit of the age‐related reference range. This case highlights the importance of examining the full laboratory profile, including both pterins and monoamine metabolites, when evaluating these patients. Here, the pterin profile is suggestive of *GCH1*‐deficiency. The mildly abnormal pyridoxal phosphate and 5‐MTHF level are of uncertain origin. For Patient 2, an elevation of BH2 would be suggestive of SRD.

^a^ Typically, SRD patients also have low HVA, 5‐HIAA. In this patient, these values were normalized on treatment with levodopa and 5‐HTP.

^b^ 5‐MTHF deficiency as seen here, is sometimes seen with levodopa treatment. Folinic acid supplementation is recommended in these patients.

Abbreviations: CSF, cerebrospinal fluid; HVA, homovanillic acid; 5‐HIAA, 5‐hydroxyindoleacetic acid; 5‐MTHF, 5‐methyltetrahydrofolate; BH2, dihydrobiopterin; BH4, tetrahydrobiopterin; SRD, sepiapterin reductase deficiency; 5‐HTP, 5‐hydroxytryptophan.

**Patient 2**, a 9‐year‐old girl, was born prematurely at 27 + 6 weeks by emergency C‐section, prompted by placenta previa and spontaneous rupture of membranes. She required neonatal intensive care unit admission for respiratory support and continued supplementary oxygen until she was 9 months old. She had early GDD (not walking or speaking at 2 years) associated with excessive fatigue and irritability. A brain MRI at 18 months showed periventricular leukomalacia (PVL) (Fig. S1). Microarray comparative genomic hybridization revealed a 1q42 duplication of uncertain significance, inherited from her asymptomatic father. Baseline neurometabolic tests were normal. At 18 months, she had microcephaly, sialorrhea, axial hypotonia, asymmetric limb hypertonia, brisk reflexes in the lower limbs, and ankle clonus. All symptoms were initially attributed to prematurity. Over time, fatigue and behavioral issues progressively worsened, with diurnal fluctuation. Support was provided at school for learning difficulties. At 7 years, she was dysarthric, had inverted feet with flat arches, action‐induced dystonia, chorea/dyskinesia, hyperreflexia, clonus in both ankles, and positive Babinski sign (Video 2, part 1).

**Video 2:** Clinical features of patient 2 before and after treatment with levodopa and 5‐hydroxytryptophan

Given her symptom progression, whole genome sequencing was performed, identifying biallelic *SPR* variants [c.751A>T p.(Lys251*); c.448A>G p.(Arg150Gly)], consistent with a diagnosis of sepiapterin reductase deficiency (SRD). Oculogyric crises were retrospectively identified from parental videos. Sinemet and 5‐hydroxytryptophan (5‐HTP) treatment led to an excellent response. At 9 years, she could walk and run independently, with good fine motor function and minimal signs of action dystonia (Video 2, part 2). There was also improvement in speech with reduced dysarthria. Over time, there was reduced frequency of anger outbursts and aggression with increasing doses of 5‐HTP. CSF neurotransmitters were not collected at time of diagnosis (because of coronavirus disease 2019 restrictions), but undertaken at 9 years (Table 1).

## Discussion

Many primary NTD can mimic cerebral palsy^5^ and other neurogenetic diseases,^6^ resulting in frequent misdiagnosis and delayed diagnosis. We report two children with primary NTD, where early symptoms were attributed to a different cause. In both children, the presence of dual pathology confounded diagnosis of the NTD.

In patient 1, dysmorphism, developmental delay, and MRI abnormalities were attributed to the chromosomal microdeletion. Although borderline intellectual disability has been rarely reported in AD *GCH1*‐deficiency,^7^ cognitive function is typically preserved.^6^ It is plausible that one or more of the other genes,^3^ encompassed within the deletion are contributing to her blended phenotype (Fig. S2). Notably, the key feature of diurnal variation was not reported by her parents, until specifically asked.

Patient 2 had typical features of SRD with infantile developmental delay, hypotonia, oculogyric crises, microcephaly, progressive fatigue, dystonia, autonomic dysfunction, and cognitive and behavioral issues.^8^ However, these were somewhat masked by her prematurity and PVL, which resulted in an initial diagnosis of diplegic cerebral palsy.

Both patients illustrate the importance of recognizing NTD red flags (Table 2),^2^, ^9^ which should prompt appropriate investigations (genetics, CSF neurotransmitters, enzyme assays) and a trial of l‐dopa.^10^ Importantly, both patients showed an excellent response to l‐dopa and/or 5‐HTP, which positively impacted their long‐term outcome. Timely diagnosis of such treatable neurotransmitter disorders is paramount, to allow early intervention and maximize the benefits of treatment.^9^

**TABLE 2:** Red flags of neurotransmitter disorders

Red flags
Diurnal variation with less severe symptoms in the morning Excess of fatigue, sleepiness Improvement of symptoms after sleep Early onset parkinsonism‐dystonia Progressive and often complex movement disorder (dystonia, parkinsonism, tremor, chorea) Oculogyric crisis Autonomic dysregulation (temperature instability, sweating, hypersalivation, nasal congestion, ptosis) Neuropsychiatric features (behavioral difficulties, irritability, anxiety) Hyperprolactinemia Good levodopa response

*Note*: Red flags suggestive of the diagnosis of a neurotransmitter disorder in children presenting with global developmental delay.

In summary, these patients illustrate the importance of keeping an “open diagnostic mind,” because premature diagnostic closure risks oversight of such treatable disorders.

## Author Roles

(1) Manuscript: A. Writing of the First Draft, B. Review and Critique; (2) Implicated in the Clinical Follow‐Up of Patients and the Recording Of Videos; (3) Implicated in the Execution/Interpretation of the Genetic Study; (4) Implicated in the Interpretation of the Neuroimages; (5) Implicated in the Interpretation of the Metabolic Studies.

A.S.V.: A1, 3

R.S.: 1B, 2

S.C.: 1B, 2

B.M.: 1B, 2

M.C.: 1B, 2

R.A.: 1B, 3

M.S.: 1B, 2

S.C.: 1B, 2

U.K.: 1B, 2

R.C.: 1B, 2

K.M.: 1B, 4

S.S.: 1B, 4

S.P.: 1B, 5

S.H.: 1B, 5

M.A.K.: 1B, 2, 3

## Disclosures

**Ethical Compliance Statement:** The authors confirm that the approval of an institutional review board was not required for this work. Ethical standards and guidelines of the UCL Institute of Child Health and Great Ormond Street Hospital were followed during all the process. The authors confirm that informed consent was obtained from all participants included in the article, in accordance with the ethical standards and guidelines of the UCL Institute of Child Health and Great Ormond Street Hospital. Consent for publication of videos was also obtained. The authors confirm that we have read the Journal's position on issues involved in ethical publication and affirm that this work is consistent with those guidelines.

**Funding Sources and Conflict of Interest:** The authors declare that there are no conflicts of interest relevant to this work.

**Financial Disclosures for the previous 12 months:** A.S.V. is the Holder of the: “la Caixa” Postgraduate fellowship (https://lacaixafoundation.org/en/postgraduate-fellowships-abroad) The rest of the authors declare that there are no additional disclosures to report.

## Supporting information


**Figure S1.** MRI Brain.
**Figure S2.** Overview of the 14q22.2‐14q22.3 microdeletion.

## Data Availability

Data sharing not applicable to this article as no datasets were generated or analysed during the current study.
