# Supplementary material for: Avoiding Premature Diagnostic Closure: Lessons from Two Children with Neurotransmitter Disorders Associated with Dual Pathology
Source: Mov Disord Clin Pract. 2024 Jul 31;11(9):1149–52. doi: 10.1002/mdc3.14164 (PMC11452789; doi:10.1002/mdc3.14164)
Supplement: Supplementary file 1 — Figure S1. MRI Brain. Figure S2. Overview of the 14q22.2‐14q22.3 microdeletion. [file MDC3-11-1149-s001.docx]

**Supplementary material:**

**Figure 1: MRI Brain:**


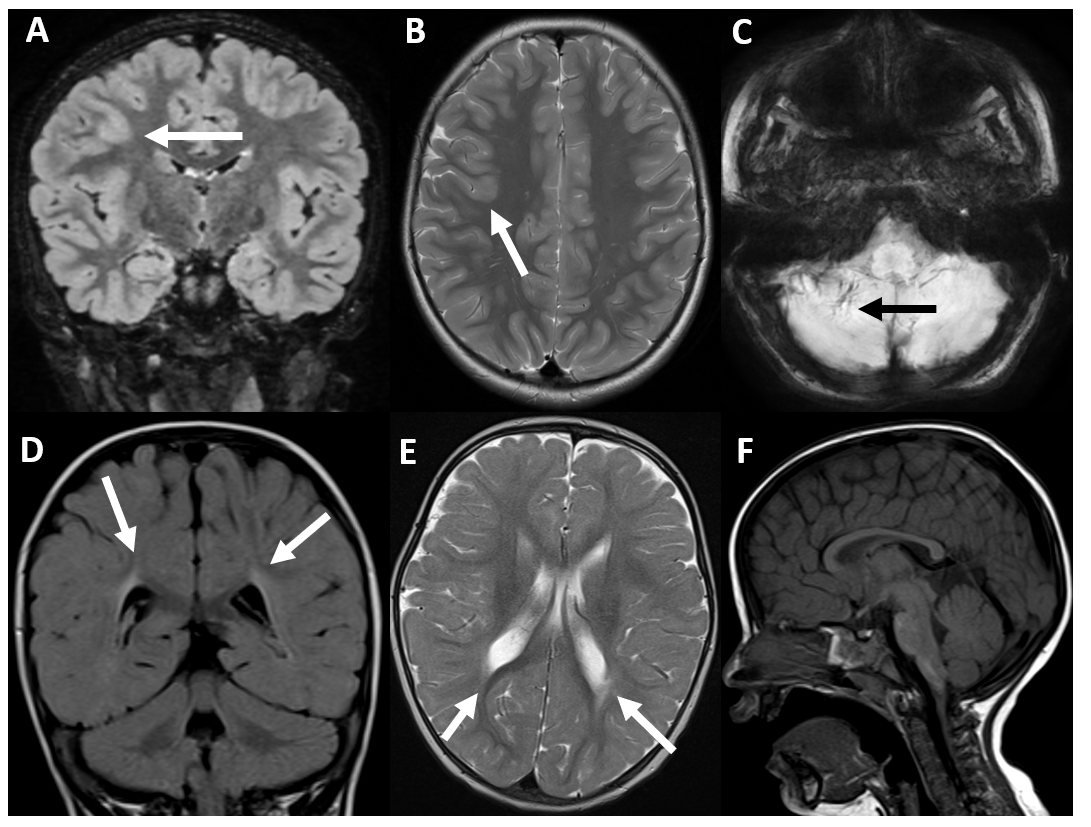


Case 1 (A, B, C): The patient was 8 years at the time of imaging. Coronal FLAIR (A), axial T2 (B) and SWI (C) showing evidence of a focal cortical dysplasia involving the right inferior frontal sulcus (white arrows) and a right cerebellar developmental venous anomaly (black arrow, C).

Case 2 (D, E, F): The Patient was 18 months old at the time of imaging. Coronal FLAIR (D), axial T2 (E) and sagittal T1 (F) showing scarring in the periventricular white matter (arrows) with consequently thin corpus callosum posteriorly, consistent with white matter disease of prematurity.

**Figure 2: Overview of the 14q22.2-14q22.3 microdeletion:**

**
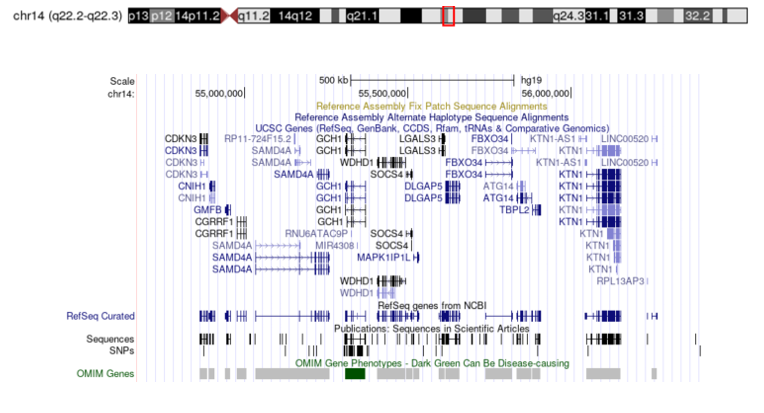
**

Schematic representation of the microdeletion region and the genes within it. Image obtained from Genome Browser ([*https//genome.ucsc.edu*](file:///C:\Users\aisa8\Downloads\https\genome.ucsc.edu)). Among the included genes, only *GCH1* has a described OMIM phenotype. *GMFB* and other genes like *MAPK1IPL1*, *KTN1*, and *ATG14* are expressed in the brain but are not currently associated with diseases. Hayashi et al. (2008)^3^ proposed *GMFB* as a candidate gene for neurodevelopmental delay, and Lim et al. (2004) ^4^  reported learning difficulties in *GMFB*-knockout mice. Given its role in glial maturation and the crucial function of glial cells in guiding developing neurons to specific cortical layers, disruptions in *GMFB* may possibly be a contributing factor to anomalies in cortical migration.
